# Synchronous thyroid and colon metastases from epidermoid carcinoma of the lung: case report

**DOI:** 10.1590/S1516-31802010000600011

**Published:** 2010-12-02

**Authors:** Carla Rameri Alexandre Silva de Azevedo, Loureno Cezana, Eduardo Sampaio Patrício de Moraes, Maria Dirley Ferreira de Souza Begnami, Tadeu Ferreira Paiva, Aldo Lourenço Abbade Dettino, Marcello Ferretti Fanelli

**Affiliations:** I MD. Resident of medical oncology, Hospital A. C. Camargo, São Paulo, Brazil.; II MD. Resident of pathology, Hospital A. C. Camargo, São Paulo, Brazil.; III PhD. Pathologist, Department of Pathology, Hospital A. C. Camargo, São Paulo, Brazil.; IV MD. Medical oncologist, Department of Medical Oncology, Hospital A. C. Camargo, São Paulo, Brazil.

**Keywords:** Thyroid gland, Lung neoplasms, Colon, Neoplasm metastasis, Carcinoma, non-small-cell lung, Glândula tireóide, Neoplasias pulmonares, Colo, Metástase neoplásica, Carcinoma pulmonar de células não pequenas

## Abstract

**CONTEXT:**

Non-small cell lung cancer (NSCLC) progresses to distant metastases in most cases. The most frequent sites for distant metastases are the bones, central nervous system, adrenal glands and liver. Dissemination to the skin, myocardium, thyroid gland and intestine may occur, but is rare.

**CASE REPORT:**

We describe a case of squamous cell carcinoma in the lungs, with metastases in the colon and thyroid, in a 66-year-old female patient. The lesion was unresectable and chemotherapy was started. The patient evolved with intestinal subocclusion, and colonoscopy showed the presence of a polyp. Biopsy and immunohistochemical analysis on the polyp showed that it was compatible with squamous cell carcinoma of pulmonary origin. At a follow-up consultation, the patient presented a thyroid nodule. A aspiration biopsy and cellblock immunohistochemistry confirmed the squamous cell carcinoma of pulmonary origin. After third-line chemotherapy, the patient progressed with acute obstructive abdomen due to a retroperitoneal mass. She underwent exploratory laparotomy and died due to surgical complications. Metastases to the thyroid and colon are rarely reported in cases of epidermoid carcinoma of the lungs. Gastrointestinal involvement in pulmonary metastases may affect the stomach, small intestine and colon, and cases of bleeding and perforation have already been reported. Although richly vascularized, the thyroid is an infrequent site for metastases. Such sites reflect poor prognoses for the clinical evolution. We did not find any previous reports in the literature, on lung cancer with metastases concomitantly in the colon and thyroid, in a single patient.

## RESUMO

**CONTEXTO:**

O câncer de pulmão de células não pequenas evolui, na maioria dos casos, com metástases a distância. Ossos, sistema nervoso central, glândula adrenal e fígado são os sítios mais frequentes de metástases. Disseminação para pele, miocárdio, tireoide e intestino pode ocorrer, entretanto é rara.

**RELATO DE CASO:**

Descrevemos um caso de carcinoma espinocelular (CEC) de pulmão com metástase em cólon e tireoide, em mulher de 66 anos. A lesão era irressecável, e foi iniciada quimioterapia. A paciente evoluiu com suboclusão intestinal e a colonoscopia evidenciou pólipo colônico cuja biópsia e imunoistoquímica (IHQ) foram compatíveis com CEC de origem pulmonar. Em consulta de acompanhamento, detectou-se nódulo tireoidiano cuja punção aspirativa e IHQ de *cellblock* confirmaram CEC de origem pulmonar. Após quimioterapia de terceira linha, a paciente evoluiu com abdome agudo obstrutivo por massa retroperitoneal, sendo submetida a laparotomia exploradora, vindo a falecer por complicações da cirurgia. Metástases para tireoide e cólon são raramente reportadas em carcinomas epidermoides de pulmão. O envolvimento gastrointestinal por metástases de pulmão pode acometer estômago, intestino delgado e cólon, e casos de sangramento e perfuração já foram reportados. Embora ricamente vascularizada, a tireoide é sítio infrequente de metástases. Os sítios refletem prognósticos reservados na evolução clínica. Não encontramos, na literatura, relato prévio de câncer de pulmão com metástases em cólon e tireoide concomitantes em um único paciente.

## INTRODUCTION

Metastatic non-small cell lung cancer (NSCLC) has a very poor prognosis. Distant metastases have most commonly been diagnosed in the brain (47%), bones (36%), liver (22%), adrenal glands (15%) and lungs (11%). Metastases in the myocardium, skin, thyroid gland and intestine may occur, but are uncommon.^1^ We report a case with synchronous thyroid and bowel metastases from squamous cell carcinoma of the lungs.

## CASE REPORT

An asymptomatic 66-year-old female was diagnosed with a mass in the lower lobe of the right lung from a screening X-ray. She had a history of non-Hodgkin's lymphoma (NHL) in 1984, which had been treated with radiotherapy and chemotherapy, and she had been undergoing follow-up since 1990. Moreover, she had chronic obstructive pulmonary disease relating to tobacco smoking for many years, and congestive heart failure. Computed tomography confirmed the lesion, and a transbronchial biopsy showed the presence of poorly differentiated squamous cell carcinoma.

The tumor had invaded the parietal pericardium, and the hilar nodes had become enlarged; thus, it was staged as IIIA. Pneumonectomy was contraindicated because of the patient's surgical risk, and combined treatment could not be performed because of the extent of the predicted radiotherapy field. At this time, the patient had already noted thyroid enlargement, and the head and neck surgeon's impression was that she presented multinodular goiter. Fine-needle aspiration biopsy was not performed, because she was going to begin oncological treatment.

**Figure 1.**
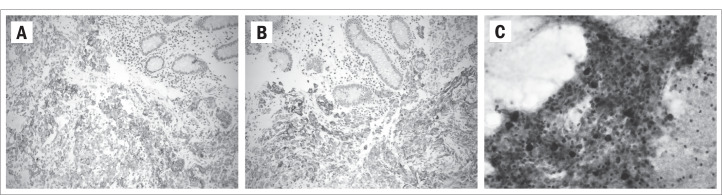
Immunostaining (x 200). A – Staining positive for CK7 in neoplastic cells in colon. B (x 200) and C (x 400) – Staining positive for 34BE12 (B – colon; C – thyroid gland).

**Figure 2.**
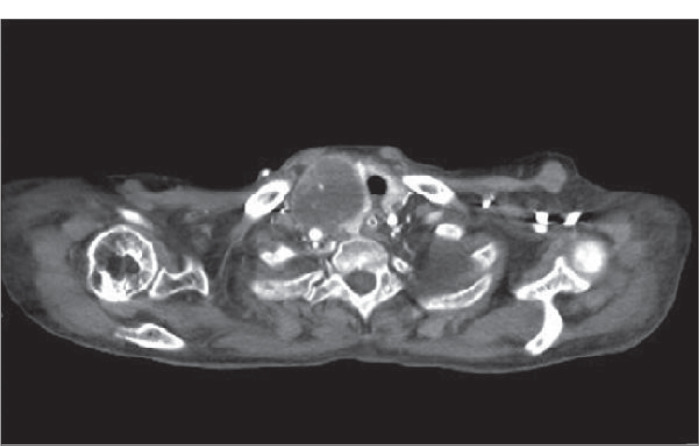
Computed tomography of thyroid mass.

**Figure 3.**
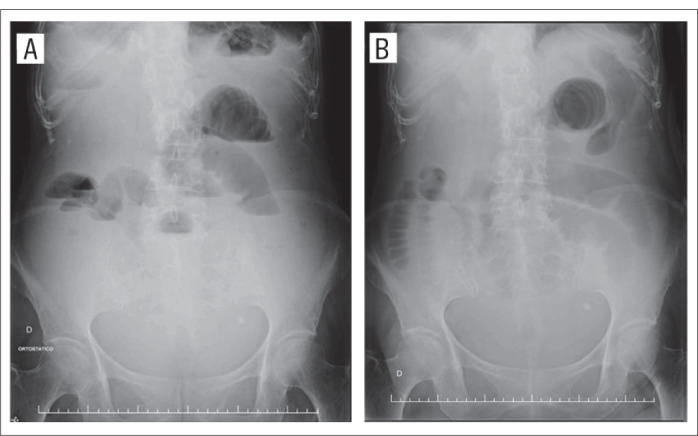
Abdominal X-ray. A and B: small bowel distention.

Induction carboplatin and paclitaxel were administered. After the second cycle, the patient presented symptoms of bowel subocclusion, subsequently followed by diarrhea. Colonoscopy showed the presence of an incidental cecal polyp, and biopsy on this polyp revealed that it was an undifferentiated malignant neoplasm. Immunohistochemical analysis showed that the polyp was AE1/AE3 (+), cytokeratin 20 (-), 34BE12 (+) and TTF-1(-), i.e. compatible with metastasis from squamous cell carcinoma of the lungs (Figure 1). Treatment with vinorelbine was started. After the eighth cycle, the thyroid nodule enlarged even further (Figure 2), and therefore an aspiration biopsy was performed, which detected metastases from squamous cell carcinoma. Treatment with gemcitabine was started. After the first two cycles, she developed a bowel obstruction (Figure 3). Exploratory laparotomy detected a retroperitoneal mass that was causing ileal obstruction, proximally to the ileocecal valve. The patient therefore underwent enterectomy and right hemicolectomy. She died from septic postoperative complications. No necropsy was performed.

## DISCUSSION

Lung cancer is frequently metastatic at the time of diagnosis. The signs and symptoms presented are often caused by the metastasis. Thyroid gland and gastrointestinal tract metastases are reported in 4% and 12% of squamous cell carcinoma cases, respectively.^2^

Lung cancer manifesting as gastrointestinal tract metastasis is exceedingly rare in clinical practice. It is a diagnostic challenge and a sign of late-stage disease. Few cases of bowel metastases from lung carcinoma have previously been reported.^3^ Cases of small bowel perforation,^4^ intussusception^5^ and bleeding^6^ have already been described. The largest series of lung carcinomas initially presenting with gastrointestinal involvement that has been described consisted of eighteen cases.^7^ The small bowel was the most common gastrointestinal site involved (12 cases), followed by the stomach (four cases) and large intestine (two cases). Immunostaining with TTF-1, CDX2, CK7 and CK20 has been found to be helpful in highlighting occurrences of primary lung adenocarcinoma. Lung cancer presenting as gastrointestinal tract metastasis is associated with dismal outcomes, and pulmonary resection coupled with chemotherapy might provide a therapeutic option for selected patients with a solitary gastrointestinal tract metastasis.^7^ However, our patient underwent emergency colectomy with palliative intent.

Cancer metastasis to the thyroid is uncommon. Although the thyroid is richly supplied with blood, there are few reports of metastatic cancer spreading to this gland. The overall incidence in autopsy series has been quite varied, with malignant tumor rates ranging from 1.2 to 24%.^8^ The most common site of origin is the kidneys. A neck mass is usually the initial presentation. The diagnosis may be made by means of fine-needle aspiration biopsy with cytological analysis, or even by means of surgery. Radiotherapy may result in disease stabilization. The majority of such patients have been found to present widespread metastases and, as a result, had very short survival times.^9^ Although detection of metastases in the thyroid gland often indicates poor prognosis, aggressive surgical and medical treatment may be effective, especially if the metastasis is an isolated occurrence. Chemotherapy is a therapeutic option for treating disseminated disease, as used in the case of our patient, with palliative intent.^8^

Distant recurrences are more frequent in patients with non-squamous lung cancer, whereas squamous cell carcinoma usually presents locoregional dissemination. This pattern of metastasis, which occurred in our patient, was unrelated to previous radiotherapy, since there was no field overlap with the NHL treatment and the primary lesion of the lung. Previous chemotherapy exposure usually does not alter the pattern of dissemination of solid tumors. In the present case, moreover, the time that had elapsed since the last treatment was as long as 20 years.

There are few data on isolated thyroid gland or bowel metastases from lung cancer (Table 1). Rarer still is the presentation of lung carcinoma with metastatic lesions in the bowel and thyroid gland at the same time. Our patient is the first reported case with concomitant pathological and clinical findings of these occurrences.

**Table 1.** Search strategies performed and results from each database

Database	Key word	Results	Relevant findings
Lilacs (Literatura Latino-Americana e do Caribe em Ciências da Saúde)	(Lung) AND (Atypical) AND (Metastasis)	4 articles	None of them demonstrated atypical metastasis from lung cancer.
	(Lung cancer) AND (Thyroid gland) AND (Metastasis)	8 articles	None of them demonstrated atypical metastasis from lung cancer.
	(Lung cancer) AND (Intestinal tract) AND (Metastasis)	7 articles	One article about small bowel intussusception due to metastatic lung cancer.
	(Lung cancer) AND (Intestinal tract) AND (Thyroid) AND (Metastasis)	0 articles	
Cochrane Library	(Lung) AND (Atypical) AND (Metastasis)	7 articles	None of them demonstrated atypical metastasis from lung cancer.
	(Lung cancer) AND (Thyroid gland) AND (Metastasis)	3 articles	None of them demonstrated atypical metastasis from lung cancer.
	(Lung cancer) AND (Intestinal tract) AND (Metastasis)	17 articles	None of them demonstrated atypical metastasis from lung cancer.
	(Lung cancer) AND (Intestinal tract) AND (Thyroid) AND (Metastasis)	1 articles	Did not demonstrate atypical metastasis from lung cancer.
PubMed	(Lung) AND (Atypical) AND (Metastasis)	364 articles	One article about thyroid metastasis that occurred 30 months after resection of an atypical bronchial carcinoid.
	(Lung cancer) AND (Thyroid gland) AND (Metastasis)	325 articles	Among these articles, we identified five case reports on thyroid metastasis from lung cancer: two adenocarcinomas, one squamous, one bronchioloalveolar carcinoma and one small-cell carcinoma. There were also four retrospective analyses on metastasis in the thyroid gland, that included 18 cases originating from lung cancer.
	(Lung cancer) AND (Intestinal tract) AND (Metastasis)	288 articles	38 articles were selected. The majority of them related to small bowel involvement, with bleeding, obstruction or perforation. There were four reports on gastric involvement and five on the colon.
	(Lung cancer) AND (Intestinal tract) AND (Thyroid) AND (Metastasis)	13 articles	None of them demonstrated atypical metastasis from lung cancer.

Last search for data in Lilacs and Cochrane Library was on April 29, 2010.

Last search for data in PubMed was between April 29 and May 1, 2010.

## References

[1] Quint LE, Tummala S, Brisson LJ (1996). Distribution of distant metastases from newly diagnosed non-small cell lung cancer. Ann Thorac Surg.

[2] Matthews MJ (1976). Problems in morphology and behaviour of bronchopulmonary malignant disease editors. Lung cancer: natural history, pognosis and therapy.

[3] Yuksel O, Uyar P, Sahin TT, Demirhan B (2007). Small bowel perforation due to metastatic lung squamous cell carcinoma.carcinoma. Saudi Med J.

[4] Listrom MB, Davis M, Lowry S (1988). Intussusception secondary to squamous carcinoma of the lung. Gastrointest Radiol.

[5] Garwood RA, Sawyer MD, Ledesma EJ, Foley E, Claridge JA (2005). A case and review of bowel perforation secondary to metastatic lung cancer. Am Surg.

[6] Pang JA, King WK (1987). Bowel haemorrhage and perforation from metastatic lung cancer Report of three cases and a review of the literature. Aust N Z J Surg.

[7] Rossi G, Marchioni A, Romagnani E (2007). Primary lung cancer presenting with gastrointestinal tract involvement: clinicopathologic and immunohistochemical features in a series of 18 consecutive cases. J Thorac Oncol.

[8] Wood K, Vini L, Harmer C (2004). Metastases to the thyroid gland: the Royal Marsden experience. Eur J Surg Oncol.

[9] Giuffrida D, Ferraù F, Pappalardo A (2003). Metastasis to the thyroid gland: a case report and review of the literature. J Endocrinol Invest.

